# Ultra-High Concentration Vertical Homo-Multijunction Solar Cells for CubeSats and Terrestrial Applications

**DOI:** 10.3390/mi15020204

**Published:** 2024-01-29

**Authors:** Ahmad A. Abushattal, Antonio García Loureiro, Nour El I. Boukortt

**Affiliations:** 1 Centro Singular de Investigación en Tecnoloxías de Información (CiTIUS), Universidad de Santiago de Compostela, 15705 Santiago de Compostela, Spain; nourelislam.boukortt@rai.usc.es; 2 Department of Physics, Al-Hussein Bin Talal University, P.O. Box 20, Ma’an 71111, Jordan

**Keywords:** GaAs solar cell, TCAD, simulation, vertical-multijunction solar cell, CubeSats, air mass 1.5

## Abstract

This paper examines advances in ultra-high concentration photovoltaics (UHCPV), focusing specifically on vertical multijunction (VMJ) solar cells. The use of gallium arsenide (GaAs) in these cells increases their efficiency in a range of applications, including terrestrial and space settings. Several multijunction structures are designed to maximize conversion efficiency, including a vertical tunnel junction, which minimizes resistive losses at high concentration levels compared with standard designs. Therefore, careful optimization of interconnect layers in terms of thickness and doping concentration is needed. Homo-multijunction GaAs solar cells have been simulated and analyzed by using ATLAS Silvaco 5.36 R, a sophisticated technology computer-aided design (TCAD) tool aimed to ensure the reliability of simulation by targeting a high conversion efficiency and a good fill factor for our proposed structure model. Several design parameters, such as the dimensional cell structure, doping density, and sun concentrations, have been analyzed to improve device performance under direct air mass conditions AM1.5D. The optimized conversion efficiency of 30.2% has been achieved with investigated GaAs solar cell configuration at maximum concentration levels.

## 1. Introduction

Day-by-day demand for solar cells is increasing due to widespread ground-based and space applications. One of the most important targets for this application is to obtain the highest efficiency to convert solar radiation to electric power. High-concentration photovoltaics are aimed at enhancing sunlight conversion efficiency and reducing the costs of electricity. CPV systems exceeding 1000 suns are called ultra-high concentrator photovoltaic systems (UHCPVs). CPVs with these technologies are considered one of the most promising avenues for achieving low cost with high efficiency. As a result of these extreme concentrations, the cell area can be dramatically reduced, which is a cost-effective way to increase the theoretical efficiency of the system [1,2,3,4] and, at the same time, to reduce the economic costs of the whole system [4,5]. Although a UHCPV has potential, it is still a work in progress, and analysis of different solar cell architectures with maximum efficiency for UHCPVs is still necessary. Obtaining high-efficiency and low-cost new-generation CPV systems is one of the chief challenges for UHCPVs with a concentration factor (C_ratio_) of more than 1000 suns. This can be explained by considering that the efficiency of solar cells grows with concentration ratio and semiconductor material costs are reduced [6,7]. In addition, photovoltaic cells are operating at ultra-high concentrations (UHCPV) for better performance. CPV systems today are commonly made up of multijunction (MJ) horizontal structures using multiple III-V semiconductors [8,9,10,11].

Multijunction fabrications based on stacking multiple solar cells can increase efficiency with a high absorption coefficient [12,13]. The direct bandgap material has proven to be highly effective in multijunction solar cells to obtain highly efficient devices for both terrestrial and space missions [14,15,16,17,18]. Also, multijunction solar cells have many advantages due to utilizing most parts of the solar spectrum, which yields high-efficiency solar cells with low power loss [19,20].

A top and a back terminal are the only electrical connections on these cells. Metal grid shadowing and series resistances (Rs) are inevitable trade-offs. Cells with a top-metal pattern or reduced cell area, which would reduce current, seem to be limited in their ability to peak at UH levels. Vertical-multijunction (VMJ) architecture reduces the Rs limitations of current architecture in an easy-to-implement manner. There are multiple sub-cells connected by a series of metallic contacts on the sides of these cells [7,11,21,22,23].

In the 1970s, vertical multijunction solar cells, otherwise known as edge-illumination solar cells, were proposed. This non-monolithic edge-illuminated junction is made up of a number of these junctions connected in parallel or series [24,25]. In such a structure, PN junctions are vertically oriented to the incident light. They have a small width and a large height. In this case, there is no shading issue and series resistance limitations are reduced. As concentration increases, efficiency increases. Compared with other power processing cells, the VMJ cell offers high voltage and low current operation. Further, its module exhibits good performance under nonuniform illumination and has a good spectral response at long wavelengths [7,25].

**Figure 1:**
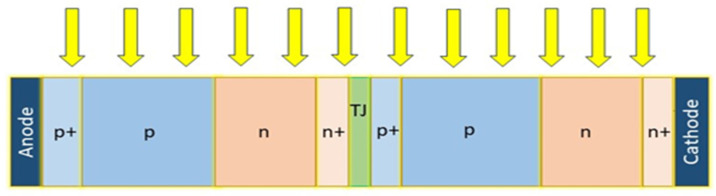
Device structure of GaAs VMJ solar cell.

In this work, the authors optimize and evaluate a novel vertical solar cell structure recently presented by Fernández, E. F. et al. with lattice-matched structures under UHCPV using physically based numerical modeling under a variety of conditions (from 1 to 10,000 suns)—these semiconductor structures have been optimized regarding their structural properties (thicknesses and doping) [21], see Figure 1. Direct-gap semiconductors or indirect bandgap semiconductors could be used with this early design. As a result, the appropriate energy gap can be selected based on the application. A single-band structure is considered in this study and an optimization procedure is conducted to determine the expected maximum efficiency of the new structure. This study lays the groundwork for understanding and evaluating the potential of this device to operate at concentration factors in potentially competitive CPV systems. We present an attempt to design an ultra-high-efficiency concentrator solar cell to help guide future concentrator solar cell manufacturing and characterization. The structure of the paper can be summarized as follows: The next section presents the role of UHCPVs, and after that, the physical background of solar cells is introduced. The following sections discuss several different device structures, as well as simulation techniques that illustrate these devices. There will be an analysis of the recombination models and simulation details, followed by the results and discussion. A conclusion and future work will be presented at the end.

## 2. The Role of Ultra-High Concentrator Photovoltaics 

In photovoltaic research, ultra-high concentrator photovoltaics (UHCPV), or solar cells exposed to 1000 sunlight concentrations, represent a crucial frontier. In concentrator photovoltaics (CPV), a high concentration is achieved through the use of lenses or mirrors. A significant improvement in efficiency achieved by this method is particularly beneficial for multijunction solar cells, which have superior efficiencies despite their high cost [1,23]. Increasing efficiency is essential to reducing material costs. Second, concentrating sunlight may reduce the amount of costly photovoltaic material needed for solar power generation, thus potentially reducing its cost and enhancing its competitiveness. A third reason is that small, highly efficient solar cells are more practical to deploy in space applications when weight and space are limited. In addition, this high-concentration approach catalyzes advances in photovoltaic material science, enabling the development of novel materials with extreme durability while maintaining efficiency and pushing the boundaries of existing technologies [23]. A high-concentration system for large-scale solar installations becomes especially attractive due to escalating global energy demands and limited land areas. As a result, investigating solar cells exposed to up to 10,000-sunlight is not just about improving efficiency but also about addressing economic viability, material innovation, and space application feasibility.

## 3. GaAs for Miniaturized Satellite and Terrestrial Application 

The efficiency of the energy conversion in solar modules for terrestrial applications is about 20%. The efficiency of the solar cell in space applications is about 30% using multijunction solar cells [26,27,28,29]. These multijunction solar cells consist of many layers of semiconductor materials to respond to the solar spectrum for more efficiency. We should try to increase the number of layers to absorb incident light. Furthermore, for space applications, we need a material that resists degradation, and gallium arsenide is one of them. Recently four or more multijunction solar cells have become a target for all researchers around the world to reach more efficiency [30,31]. Gallium arsenide (GaAs) is a III–V compound. It consists of both gallium and arsenic from the third (III) and fifth (V) columns of the periodic table [32]. GaAs is commonly used in space and military applications because it has advantages such as the direct band gap and high resistivity to radiation and heat [33,34]. Furthermore, it permits electrons to move faster, about six times, than silicon substrate does and works at higher frequencies [35]. 

CubeSats are miniaturized satellites that use this GaAs semiconductor. Several properties make it suitable for CubeSats: Due to its high electron mobility and breakdown voltage, it can withstand high voltages without degrading [35,36]. Therefore, it can be used to power electronic devices such as solar cells, which convert solar energy into CubeSat power. The material is thermally stable, meaning that it is capable of withstanding high temperatures without deteriorating. When CubeSats are launched and operated in space, they might be exposed to high temperatures. The energy band gap of GaAs, compared with silicon, gives it the ability to generate more energy from light [37,38]. As a result, it is a good material for use in solar cells, which are used to power CubeSats, and it is relatively light in weight. In several CubeSat missions, GaAs solar cells have proved to be efficient and reliable solutions for power generation. GaAs is relatively expensive and is only used for specific applications where the benefits outweigh the costs. One example of a CubeSat that uses GaAs technology is the NASA Ames Research Center’s PhoneSat 2.5. It is a 10 × 10 × 11.35 cm CubeSat weighing 1.5 kg. It was launched in 2013 as PhoneSat 2.5 [39,40]. The Japan Aerospace Exploration Agency (JAXA) proposed a CubeSat-based solar power satellite (CS-SPS). CubeSats, or miniature satellites, serve a variety of purposes and can be placed in various locations based on their missions [40,41]. The low earth orbit (LEO) uses CubeSats for a variety of missions, including Earth observation, remote sensing, and scientific research. CubeSats are often used for navigation and communication purposes [42,43]. Medium earth orbit CubeSats (MEO) fly between 2000 and 35,786 km above the surface of the Earth [44]. At an altitude of 35,786 km, geostationary earth orbit CubeSats (GEOs) are used for communication and remote sensing. In lunar orbit, CubeSats conduct scientific research on the Moon and explore its surface. Several interplanetary missions have used CubeSats, such as Mars Cube One (MarCO). CPV systems use GaAs solar cells to concentrate sunlight onto high-efficiency solar cells, which use lenses or mirrors to focus sunlight. As GaAs solar cells have high thermal stability and can operate at high temperatures, they are well suited for use in applications involving high temperatures, such as desert power plants. In addition to being highly reliable and high-performing, GaAs solar cells have a long lifetime and high efficiency. GaAs solar cells are widely used for developing new solar cell technologies and testing the limits of their efficiency and stability in research and development. Due to their high efficiency and high-temperature performance, GaAs solar cells are limited to specific applications where their cost does not outweigh their benefits. Cubesats and satellites can be powered by AM1.5 solar cells to provide power to their onboard systems [45,46,47]. The amount of electrical power the cells can generate is determined depending on the efficiency of the solar cells, which can be measured under AM1.5 conditions. Cubesats and terrestrial applications need more electrical power to operate, which can be generated by higher efficient solar cells under AM1.5 conditions [47,48].

## 4. Device Structure and Simulation Technique 

This work focuses on optimizing and evaluating a recent novel vertical solar cell structure [7,9,11,21,22,49]. This early design made it possible to use direct or indirect bandgap semiconductors. It is, therefore, possible to choose an energy gap based on the application. By optimizing electrical parameters, we examine the expected maximum efficiency of a single-band structure. Additionally, we investigate how the concentration ratio affects the device’s performance. As a result of the findings of this study, a novel generation of competitive CPV systems is expected to be developed. In this study, a novel ultra-efficient concentrator solar cell will be designed, and its manufacture and characterization will be based on this research [11,50]. We study an edge-illumination multijunction solar cell consisting of non-monolithic vertical junctions connected. A PN junction receives incident light in a vertical direction. Each sub-cell has metal contacts on its lateral sides. They have a small width and a large height. Therefore, there is no shading problem or series resistance issue. The VMJ is compatible with most power supplies since it is a high-voltage, low-current cell. In this article, the authors optimize a gallium arsenide (GaAs) structure as a vertical tunnel-junction (VTJ) cell consisting of laterally positioned anode and cathode with two identical sub-cells with four layers each, which are connected by a tunnel junction (TJ), as shown in Figure 1.

We have considered using only GaAs for the TJs of VTJ cells at this stage. In this way, the structure matches the lattice and can be monolithically grown without causing problems with mismatching. There are two identical sub-cells in the vertical tunnel junction (VTJ) cell. Each sub-cell has four layers, and they are connected by a tunnel junction (TJ). The anode (A) and cathode (C) are positioned laterally. We have changed the thickness (width) and doping values of the p^+^ and n^+^-layers at this stage of concentration from one sun to ten thousand suns. An n^+^/p^+^ GaAs tunnel junction has a 25 nm width and is doped with 7 × 10^19^ cm^−3^. A direct tunneling model has been included as well as a trap-assisted tunneling model to simulate this element. p^+^ and n^+^-layers have a width of 0.07 µm and a constant doping value of 5 × 10 cm^−3^. In Figure 1, we can see the basic structure and dimensions of a vertical tunnel-junction GaAs solar cell. This material was chosen due to its high conversion efficiency. The proposed structure consists of two identical sub-cells connected by tunnel junctions. Each sub-cell is composed of two consecutive P-layers, the first one highly doped to 5 × 10^19^ cm^−3^ and the second layer to 2 × 10^17^ cm^−3^, followed by two N-layers, doped to 4 × 10^16^ cm^−3^ and 5 × 10^19^ cm^−3^, respectively. Both layers of the TJ are 25 nm wide, and both are doped to 7 × 10^19^ cm^−3^. A total of 9.73 µm in width and 1.25 µm in height make up the structure. Any value of H can be increased without affecting the performance of the cell to reach the desired height. Additionally, this structure reduces the shadowing of the front contacts and the Rs of HSCs by eliminating the trade-off between these two factors. Due to this, it is possible to also decrease the Rs losses by increasing the height and, therefore, by lengthening the lateral metallic contacts. There are only two electrical terminals needed to extract the generated current from the 1-VTJ solar cell (indicated in dark gray color in Figure 1) since it is oriented perpendicular to the sunlight. A PN junction is illuminated perpendicularly to these junctions using a reference spectrum of AM1.5D under 1000 W/m^2^ at a 25 °C cell temperature (Standard Test Conditions, (STC)). Furthermore, the reflection, heat effects, and the third dimension are negligible in this simulation.

**Figure 2:**
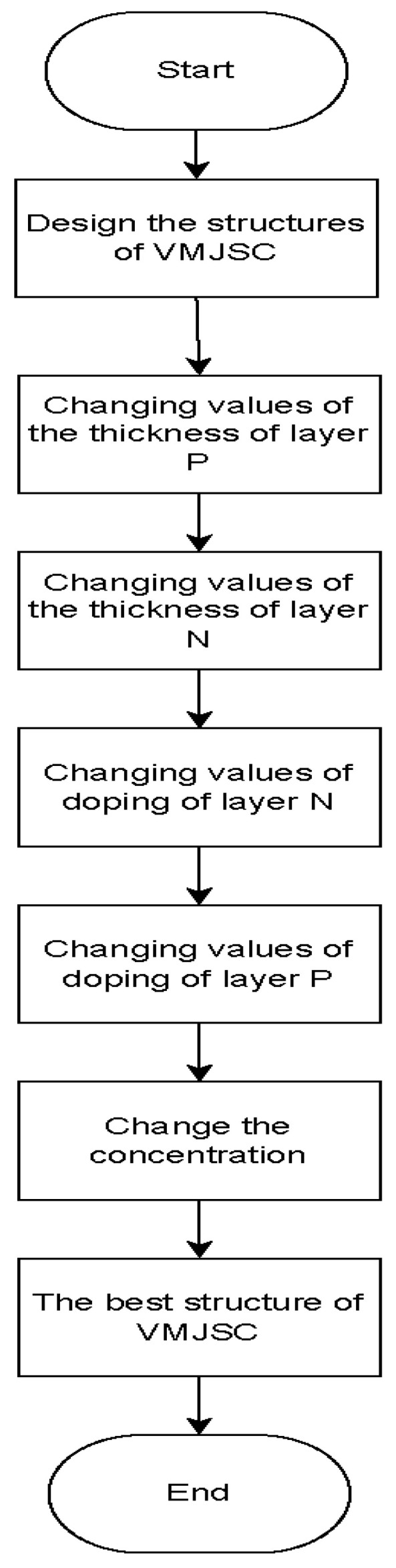
A flowchart of the methodology used to obtain the best structure of VMJSC.

Silvaco ATLAS is a state-of-the-art semiconductor simulator for photovoltaic devices. Multi-bandgap concentrator solar cells can be designed and improved with this software due to its realistic and trustworthy results. Also, as a framework, it permits us to simulate the thermal, optical, and electric behavior of semiconductor devices considering two and three dimensions. ATLAS uses the physical structure of the process simulator Athena as an input file to predict all information and characteristics of the solar cell, and each of them was developed by SILVACO [2,51,52,53]. We used the Silvaco ATLAS simulator to obtain the best structure of MJSC GaAs. We achieved several parameters: current density (J_SC_), open-circuit voltage (V_oc_), fill factor (FF), and conversion efficiency(η), among others. Figure 2 shows the algorithm of our work to obtain the electric characteristics of MJSC GaAs. In this work, we use Silvaco TCAD to construct the multijunction solar cell and then Silvaco to improve efficiency.

## 5. Recombination Models and Simulation Details

The ability to obtain highly efficient cells is critical to both terrestrial and space missions. Nowadays, technology computer-aided design (TCAD) is one of the most common platforms widely used to improve photovoltaic device (PVD) technology. This platform offers an environment to predict and explore the performance of multijunction solar cells. In this work, we used Silvaco ATLAS, a well-known TCAD tool, to investigate, simulate, characterize, and analyze ultra-high concentrations of homo-multijunction GaAs for terrestrial applications [51]. As well as providing significant contributions to the field of photovoltaic technology, this study emphasizes how important it is to continue exploring material science, cell design, and integrating solar cells with energy storage systems so that renewable energy solutions and space technology can advance. Silvaco ATLAS TCAD solved a set of fundamental equations that operate photovoltaic devices. These equations are included by default in ATLAS and are used to build the device simulator by combining the carrier densities with electrostatic potential derived from Poisson’s equation, Maxwell’s laws, carrier continuity equations, and transport equations. Poisson’s equation describes the electrostatic potential of electric charge density distribution:

(1) $$div\left(\varepsilon \nabla \varphi \right)=-\rho$$

where ε is the permittivity, φ is the electrostatic potential, and ρ is the charge density.

Carrier continuity and transport equations express electron and hole densities generation, transport, and recombination processes [53,54]:

(2) $$\frac{\partial n}{\partial t}=\frac{1}{q}div\;J_{n}+G_{n}-R_{n}$$

(3) $$\frac{\partial p}{\partial t}=\frac{1}{q}div\;J_{p}+G_{p}-R_{p}$$

where n and p are the electrons and holes concentration, J is the current densities (A/m^2^), G is the generation rates, R is the recombination rates, and q is the charge on an electron.

The recombination and carrier mobility of the vertical homo-multijunction were calculated using three recombination models: Shockley–Read–Hall (RSRH), Auger (R_Auger_), and optical generation radiative (R_rad_). Silvaco ATLAS extracts several important parameters to estimate the solar cell’s performance: short-circuit current (I_sc_), open-circuit voltage (V_oc_), and fill factor (FF). The short-circuit current (I_sc_), or (J_sc_) as a density, describes the maximum current from a solar cell, which occurs at zero voltage, where I_L_ = light-generated current.

(4) $$I_{sc}=I_{0}\left[e^{\left(\frac{qV}{nkT}\right)}-1\right]-I_{L}$$

The open-circuit voltage (Voc) is the maximum voltage at zero current.

(5) $$V_{OC\;}=\frac{\mathrm{nKT}}{q}\times \ln\left(\frac{I_{L}}{I_{0}}\right)+1$$

By comparing the maximum power with the theoretical power, it would generate at both the open-circuit voltage and short-circuit current together, the fill factor (FF) is calculated as a performance measure of the solar cell.

(6) $$\mathrm{FF}=\frac{I_{\mathrm{mp}\;}\times {\;V}_{\mathrm{mp}\;}\;}{I_{\mathrm{SC}\;}\times {\;V}_{\mathrm{OC}\;}}$$

All previous parameters yield to calculate the maximum power.

(7) $$P_{\max\;}=J_{\mathrm{SC}\;}\times {\;V}_{\mathrm{OC}\;}\times \mathrm{FF}$$

The fill factor (FF) of solar cells is calculated by Silvaco using simulations and analyses under specific conditions. Materials, layers, and doping profiles of a solar cell are initially defined by the user. In simulations, boundary conditions, such as illumination and electrical parameters, are applied to simulate real-world operating scenarios. Solving complex semiconductor equations determines the current–voltage characteristics of solar cells. This I-V curve can be used to determine key circuit parameters like the open-circuit voltage and short-circuit current. In an I-V curve, the maximum power point is the point where the product of current and voltage reaches its maximum (P_max_). Based on the formula FF = P_max_/(V_oc_ × J_sc_), the fill factor measures the efficiency of the solar cell.

Then we can calculate the efficiency of the solar cell by comparing the output–input power.

(8) $$\eta =\frac{P_{\mathrm{out}\;}\;}{P_{\mathrm{in}\;}\;}$$

(9) $$\eta =\frac{J_{\mathrm{SC}\;}\times {\;V}_{\mathrm{OC}\;}\times \mathrm{FF}}{P_{\mathrm{in}\;}\;}$$

## 6. Optimization Procedure

To verify the accuracy of our model, we must first compare it with similar cells of well-known characteristics and their resulting performance [3]. Initially, the purpose of this work was to construct a solar cell and then improve its performance. The following section describes the optimization procedure for the structure shown in Figure 1, while Table 1 describes the design and material properties. A twofold procedure will be used in this experiment: varying the intrinsic parameters (the thickness from 3 to 8 µm and the doping from 10^12^ to 10^17^ cm^−3^) and changing the extrinsic parameters (the concentrations from 1 to 10,000 suns), while keeping the thickness values of each layer unchanged throughout the simulation at 15 µm. As a starting point, we will use the values we obtained from previous work [4] for each parameter. To determine the appropriate values corresponding to the thickness and doping of the layers of the P- and N-layers, the concentrations will be kept constant during the first part of the simulation, while the intrinsic parameters of the layers will be varied to discover the most optimal values. The second part of the process consists of changing the concentration and repeating all the previous steps. It would be best to start by optimizing the thickness of the P-layers by changing their value to maximize efficiency while ensuring that the thickness of the N-layers and the doping of each P- and N-layer stay constant at the same time. After selecting the best thickness of the P-layers, we will proceed to change the thickness of the N-layers until we reach the best value, keeping the doping of the layer’s constant throughout the process until we reach the best value for the thickness of the N-layers. To change the doping on the P-layer, we selected the best values for the N- and P-layers; then, we changed the doping on the P-layer while keeping the doping on the N-layer constant to obtain the best value. Then, change the value for the N doping to reach the best value that can be achieved. After the process of changing concentrations is completed, we select a new concentration between 1 and 10,000 suns to determine the best structure for our design.

## 7. Impact of Absorber P-Layer Thickness

Solar cells are highly efficient when the front layer thickness is sufficient to generate photocurrent. The thickness of the sensitizer or absorber layer can also affect the efficiency, Jsc, Voc, and FF of the multijunction solar cell. Short-circuit current density increases with a decrease in the front layer thickness. This increases the space charge region’s collection efficiency, resulting in a greater short-circuit current density. Furthermore, when surface recombination is considered, the depletion region has weakened collection efficiency due to its proximity to the surface. The thickness of PSC greatly influences its performance. PV performance is directly affected by the absorber layer thickness, which directly affects charge carrier diffusion lengths. Charge carriers may recombine when the absorber layer is too thin, resulting in the incident radiation not being fully absorbed. Overly thick absorbers may cause charge carriers to recombine before reaching the electrodes.

The thickness of the P-type layer in a solar cell can have an impact on the efficiency, open-circuit voltage, fill factor, and short-circuit current of the cell. Increasing the thickness of the P-type layer can increase the open-circuit voltage by increasing the built-in potential across the P–N junction. However, increasing the thickness of the P-type layer can also decrease the short-circuit current because of the decreased number of carriers (electrons and holes) that are generated in the cell. The fill factor is the ratio of the maximum power output of the cell to the product of the open-circuit voltage and the short-circuit current. It is a measure of how efficiently the cell converts light into electricity. The optimal thickness of the P-type layer for the highest fill factor depends on the material system used, but usually, a thinner P-type layer gives a better fill factor. The overall effect on the efficiency of the solar cell depends on the balance between the increased Voc and decreased J_sc_. The optimal thickness of the P-type layer will depend on the specific material system, and the design of the solar cell and is typically determined through experimentation and optimization.

**Figure 3:**
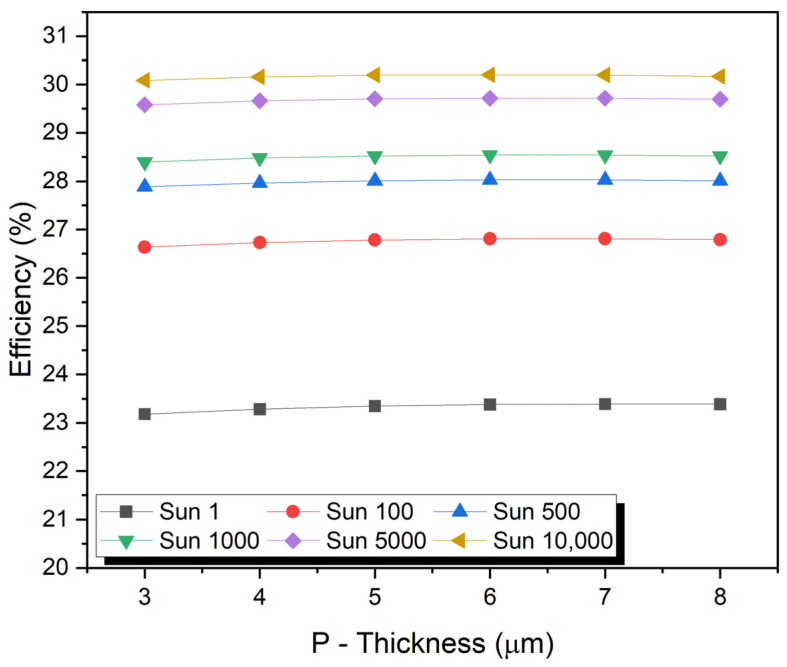
Impact of absorber P-layer thickness.

The thickness of the P-type region varied from 3.0 µm to 8.0 µm in an N- and P-type region with optimized doping concentrations of 9 × 10^15^ cm^−3^ and 2 × 10^16^ cm^−3^, respectively. The maximum values of J_sc_ of 2.45 mA/cm^2^ and V_oc_ of 1.86 V under a concentration of one sun to 24,593.5 mA/cm^2^ and V_oc_ of 2.33 V with a concentration of 10,000 suns were obtained at 2.1 µm thickness of the N-type region (see Figure 3). However, the FF and efficiency initially increased to a maximum value and then decreased. Recombination became faster as the P-region thickness increased. As the thickness of the N-type region increased, short-circuit current density and open-circuit voltage increased, according to the nature of the solar cell.

## 8. Impact of Absorber N-Layer Thickness

An absorber layer can improve PV performance, which is usually determined by the film quality and morphology of the absorber layer. It is important to consider the thickness of the absorption layer and defect density to achieve good performance parameters. This section examines absorber N-layer thickness (N_t_). A thicker N-type layer will increase short-circuit current (J_sc_) by increasing the number of carriers (electrons and holes) in the cell. Thicker N-type layers can also decrease the open-circuit voltage because the increased surface area can increase carrier recombination. The fill factor measures the power of a cell by combining the open-circuit voltage and short-circuit current. 

**Figure 4:**
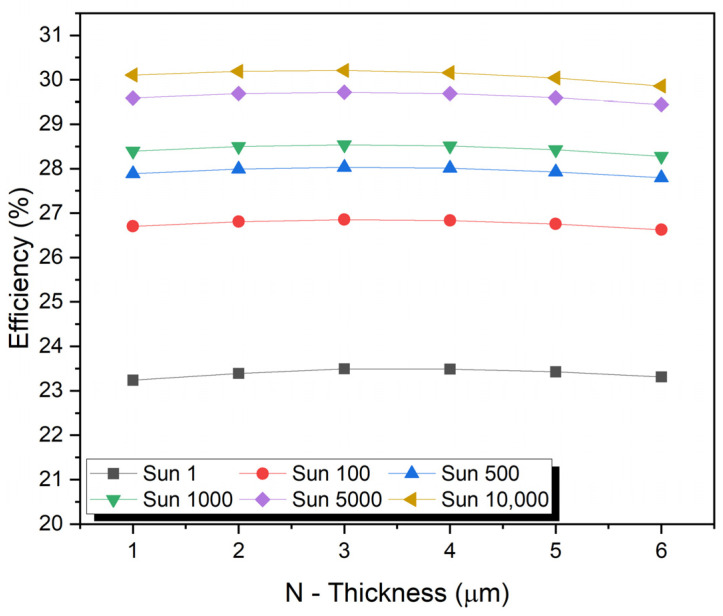
Impact of absorber N-layer thickness.

The conversion efficiency of the light into electricity depends on the material system used—whether thicker N-type layers give a higher fill factor. The efficiency of a solar cell depends on how well J_scf_ and V_oc_ balance each other. Based on the material system and design of the solar cell, the optimal thickness of the N-type layer is determined through experimentation and optimization. The thickness of the N-type region varied from 1.0 µm to 6.0 µm doping the N- and P-type regions at an optimized concentration of 9 × 10^15^ cm^−3^ and 2 × 10^16^ cm^−3^, respectively. The maximum values of Jsc of 3.23 mA/cm^2^ and V_oc_ of 1.86 V under a concentration of one sun to 29,812.1 mA/cm^2^ and V_oc_ of 2.34 V with a concentration of 10,000 suns were obtained at 7.0 µm. Figure 4 shows the thickness of the N-type region. Increasing the thickness of the N region increased the rate of recombination. The density of short-circuit currents and open-circuit voltages increased with increased thickness of N-type regions. Contrary to this, the FF and efficiency initially increased to a maximum value and then increased.

## 9. Impact of Absorber P-Layer Doping

The doping level in the P-type semiconductors affects the width of the depletion region and the built-in potential. A wider depletion region and a higher built-in potential, which can lead to better separation of the electrons and holes created by sunlight, result in a higher short-circuit current. P-doping is an important factor in designing more efficient solar cells because it creates the P-side of the P–N junction, which is essential for the separation of the created hole and electron during sunlight absorption. Increasing the concentration of free electrons in P-type material can increase J_sc_. This can cause a reduction in the built-in potential of the P–N junction, which can negatively affect the fill factor by lowering V_oc_. A high fill factor indicates that a solar cell can maintain a high level of power output over a wide range of operating conditions. 

The application of a higher dose of dopant to the P-type material leads to a higher current flow, which can enhance the fill factor. Although excessive doping may reduce the fill factor by recombining electrons and holes, excessive doping can cause recombination. P-type material can be doped both positively and negatively to increase or decrease the efficiency of a photovoltaic cell, which is determined by the interdependence of Jsc, Voc, and the fill factor. Therefore, the effect of doping P-type material on efficiency depends on how the solar cell is designed. Excessive doping, however, can cause electrons and holes to recombine, resulting in decreased efficiency. The optimal doping level is contingent upon the specific material and the design of the solar cell.

**Figure 5:**
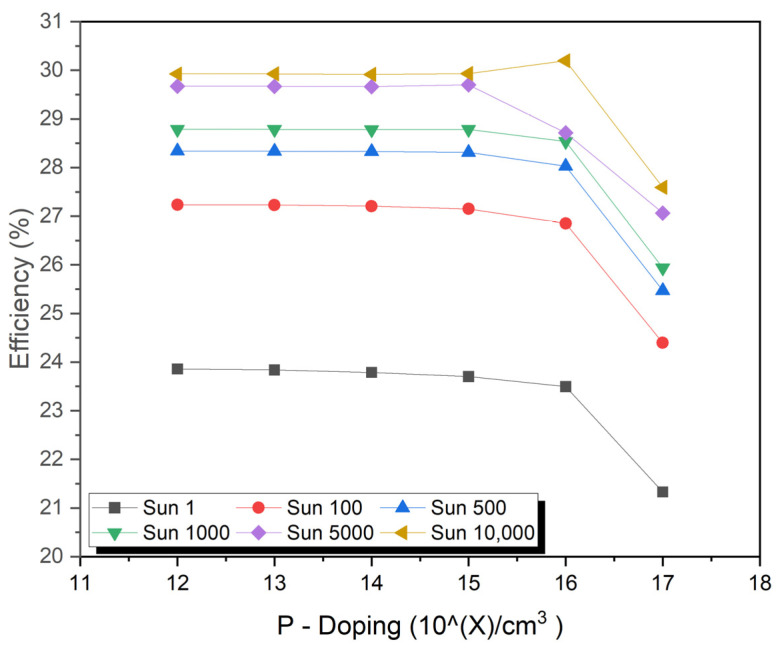
Impact of absorber P-layer doping.

The doping concentration varies from 9 × 10^12^ to 9 × 10^17^ cm^−3^ for the P-type region, as shown in Figure 5. The thickness of the P-type region is 7.0 µm, and that of the N-type region is 5.0 µm. For the N-type region, the doping concentration is optimized at 2 × 10^16^ cm^−3^. The maximum J_sc_, and V_oc_ values under a concentration of one sun are 2.74 mA/cm^2^ and 2.1 V, while under a concentration of 10,000 suns are 27,438.2 mA/cm^2^ and 2.34 V, respectively.

## 10. Impact of Absorber N-Layer Doping

**Figure 6:**
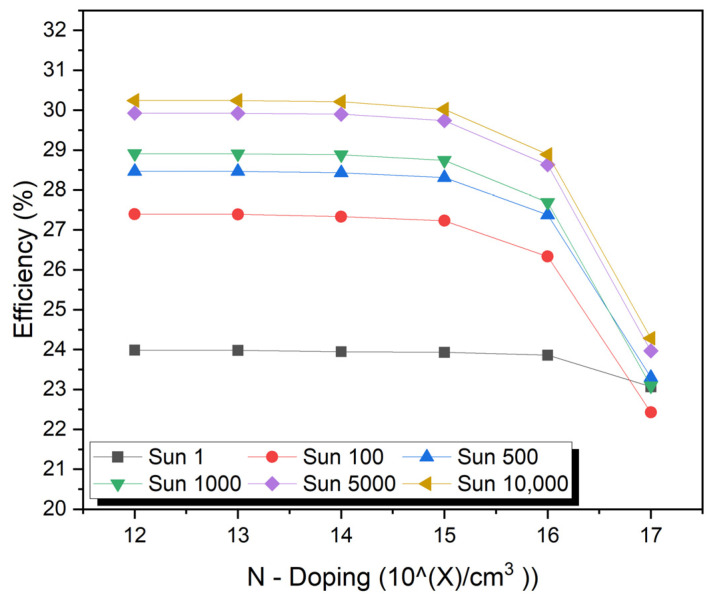
Impact of absorber N-layer doping.

Adding N-type materials to the solar cell not only improves its conductivity but also changes its electrical properties. The short-circuit current may increase as a result of the improved conductivity of the material. Additionally, the open-circuit voltage may decrease due to the reduced built-in potential of the P–N junction. By reducing the open-circuit voltage, the fill factor may decrease. Due to the reduction in both J_sc_ and V_oc_ and the effect on the fill factor, the overall efficiency of the solar cell may decrease. It will, however, depend on the specific doping conditions and material properties. The doping concentration varies from 2 × 10^12^ to 2 × 10^17^ cm^−3^ for the N-type region, as shown in Figure 6. The thickness of the P-type region is 7.0 µm, and of the N-type region, it is 5.0 µm. The P-type region should be doped at an optimal concentration of 9 × 10^16^ cm^−3^. The maximum values of J_sc_ of 2.75 mA/cm^2^ and V_oc_ of 1.86 V under a concentration of one sun to 24,715.5 mA/cm^2^ and V_oc_ of 2.33 V with a concentration of 10,000 suns have been obtained.

## 11. Influence of Concentrations

Multi-bandgap concentrator solar cells have been designed using Silvaco ATLAS, an electronic device simulator proven to provide reliable results [53,55]. In the number of suns concentration, the incoming light intensity is compared to the intensity of sunlight at the Earth’s surface (1 sun). J_sc_, V_oc_, fill factor, and efficiency are related to the number of sun concentrations as follows: The short-circuit current increases as the concentration of light increases, allowing a higher output power. As the intensity of light goes up, more electron–hole pairs are generated, which could boost the open-circuit voltage in solar cells. However, when the concentration increases, the fill factor might drop due to a rise in resistance and a potential decrease in V_oc_. Overall, the efficiency of solar cells can improve with higher concentration, but this depends on the specific design and materials of the cells. It is a balancing act between gaining more current and possibly losing some fill factor.

The temperature of the cell, the material used, and the quality of the interfaces all contribute to the efficiency, V_oc_, FF, and J_sc_ of the solar cell. Moreover, most solar cells are designed to operate under the standard test conditions (STC), which are 1000 W/m^2^ illumination and 25 °C temperature, and the efficiency is reported under these conditions. 

**Figure 7:**
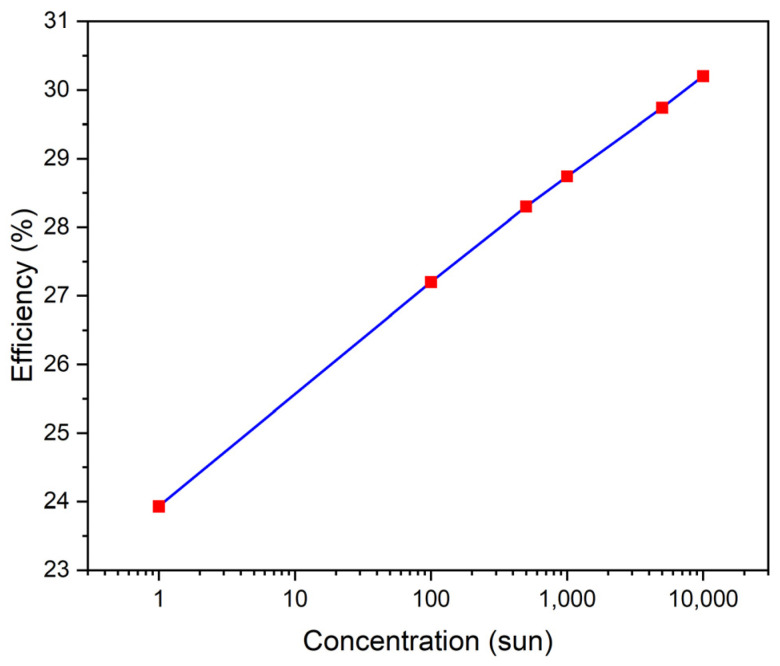
The efficiency vs. the concentration of sunlight varied from 1 to 10,000 suns.

**Figure 8:**
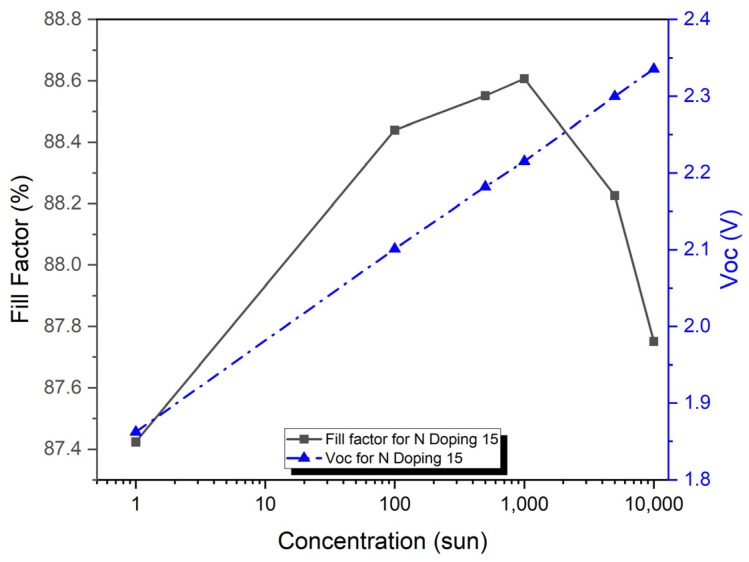
Influence of concentrations.

The concentration of sunlight varied from 1 to 10,000 Suns. The doping concentration of the P-type region is 2 × 10^16^ cm^−3^ and it is 2 × 10^15^ cm^−3^ for the N-type region. The thickness of the P-type region is 7.0 µm, and of the N-type region, it is 5.0 µm. The maximum values of J_sc_ of 2.75 mA/cm^2^ and V_oc_ of 1.86 V under a concentration of one sun to 24,715.5 mA/cm^2^ and Voc of 2.33 V with a concentration of 10,000 suns have been obtained. The efficiency is 23.93% for (one sun), 27.2% (100 sun), 28.3% (500 sun), 28.74% (1000 sun), 29.74% (5000 sun), and 30.2% (10,000 sun) as shown in Figure 7 and Figure 8.

## 12. The Combination Structure

**Figure 9:**
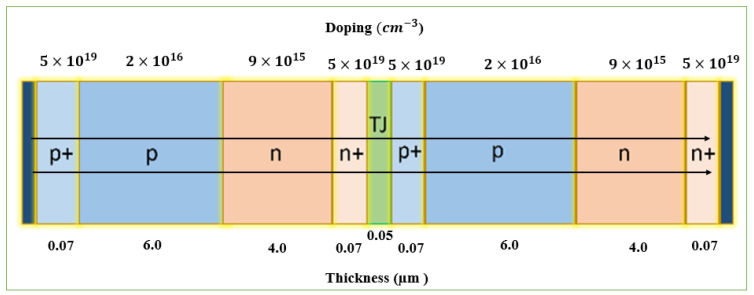
An illustration of the best structure of a VTJ solar cell.

This study optimizes the design parameters for ultra-high concentration vertical GaAS multijunction solar cells. This includes layer thicknesses, doping concentrations, and the concentration of solar radiation to maximize the efficiency of the device. Simulations can help optimize the design of a structure and compare its performance against other structures to determine which is the most appropriate for a specific application. The best structure will also depend on the materials available and the manufacturing processes available to produce the solar cell. Figure 9 shows the best values for the doping and the thickness used in this study.

The overall structure has a width (W) of 20.33 μm and a height (H) of 1.25 μm. In any case, you can increase the H to a larger value to obtain the desired height of the cell without impacting performance. Therefore, we can also increase H and, thus, the lateral metal contact length to reduce Rs loss without limit.

**Figure 10:**
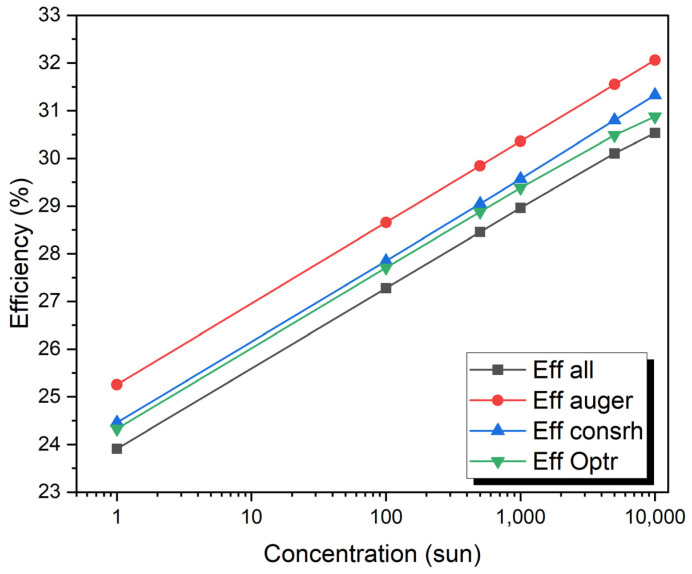
VTJ solar cell open-circuit voltage as a function of the concentrations with recombination mechanisms and with all recombination VTJ solar cells.

Figure 10 shows the V_OC_ as a function of concentration for the VTJ solar cell, showing how each recombination effect (Auger, SRH, and radiative) contributes individually and together (all recombination). V_OC_ concentration increases linearly with a logarithmic increase in all recombination mechanisms. When all mechanisms are considered, the voltage ranges from 1.86 volts at 1 sun to 2.4 volts at 10,000 suns. When all recombination effects are present, the V_OC_ is reduced by 3.45% (for 1 sun) and by 2.13% (for 10,000 suns). Up to 100 suns, the V_OC_ is also affected by the SRH and radiative effects. It is the radiative effect that degrades the V_OC_ the most at larger sun concentrations, whereas the SRH effect is insignificant at these concentrations. Cell performance is dominated by radiative mechanisms at higher concentrations, while SRH recombination mechanisms are more significant at lower concentrations. 

**Figure 11:**
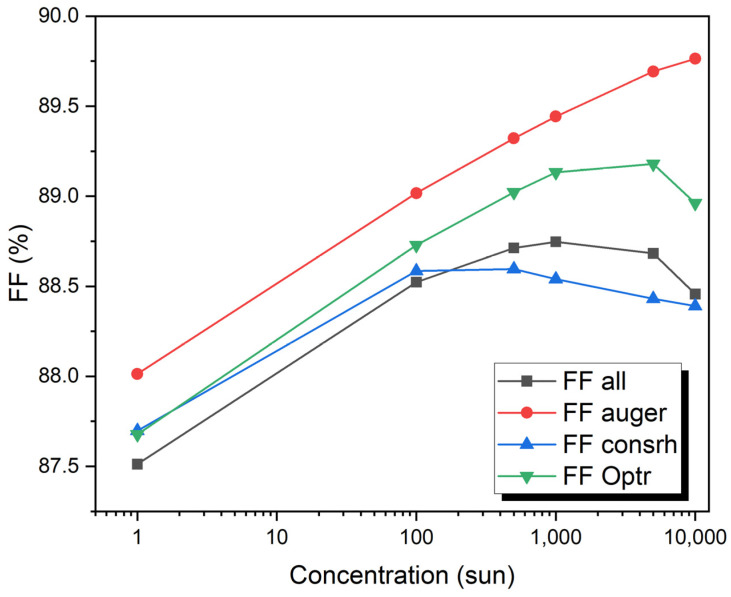
VTJ solar cell fill factors for each recombination mechanism and for all recombination.

Figure 11 shows the combined Auger, radiative, and SRH contributions of the VTJ solar cell as a function of concentration, along with their recombination (combined effects). It shows the increase of the Auger concentration as the FF increases with the logarithm. Although FF grows with concentrations up to 1000 suns for radiative, SRH, and all recombination cases, it tends to decrease for higher irradiances. When all mechanisms are considered, it ranges from 87.5% at 1 sun to 88.5% at 1000 suns. As the number of suns increases, due to R_S_ losses, the FF decreases slightly, eventually dropping to 88.3% at 10,000 suns. Even so, FF only shows a slight difference, about 1%, at all concentrations investigated. Currently, ultra-high concentration vertical homo-multijunction solar cells have low efficiency due to their small R_S_ values, which are approximately one order of magnitude below those of conventional concentrator solar cells.

**Figure 12:**
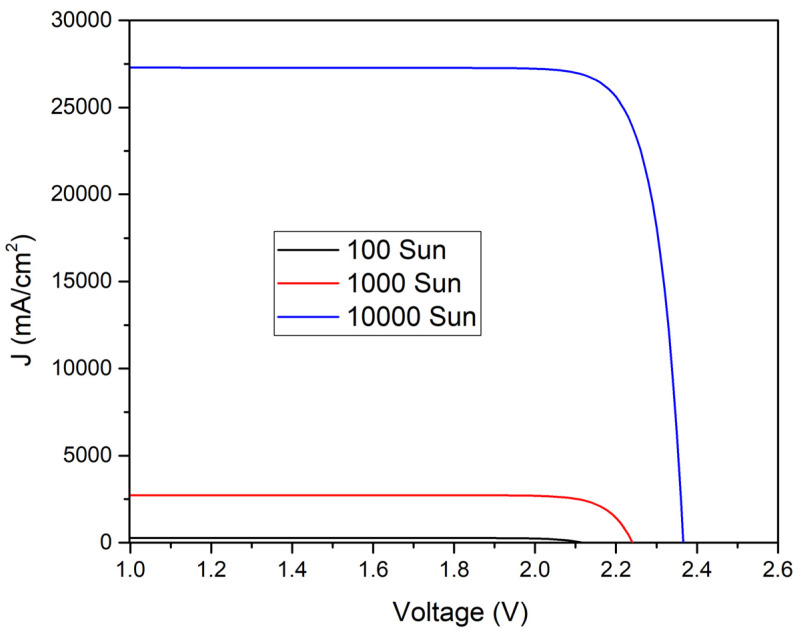
Solar cell I-V characteristics at different light intensities.

Each curve in Figure 12 shows the I-V characteristics of a solar cell under varying solar concentrations from 1 sun to 10,000 suns. As light intensity increases, key parameters like the short-circuit current and open-circuit voltage increase as well, indicating that the cell generates more power as it receives more light. Curve flatness corresponds to maximum current, while intersection points on the axes correspond to maximum voltage and current. An important factor in determining the efficiency of a cell is the area under the curve. Photovoltaic systems must be designed and optimized to maximize their performance with sunlight intensity, as shown in the graph.

## 13. Conclusions and Future Work

In this work, we present a new architecture for solar cells under concentrations of 1–10,000 suns. Its basic structure consists of two identical cells connected by a tunnel junction (TJ). This vertical tunnel-junction (VTJ) solar cell offers a unique opportunity to use highly efficient III-V direct bandgap semiconductor materials at extremely high sun concentrations. We investigate this structure of non-monolithic vertical connections at several sun concentrations. Light strikes a PN junction in a vertical direction. Metal contacts can be seen on the lateral sides of each sub-cell. Therefore, there are no issues with series resistance or shading. Because of this, cells with low current densities and low Rs values can be created, leading to ultra-high flux cells having those properties. Efficiency rises with the concentration, even as concentrations increase from 1 to 10,000 suns. VTJ structures have an advantage compared with current-collecting solar cells. As light intensity increases, we can theoretically increase efficiency by almost eliminating losses due to series resistance. On the other hand, using direct bandgap materials allows us to design multi-bandgap structures. It absorbs the spectrum better. All these advantages make this structure suitable for CubeSats and terrestrial applications. The following areas could be explored in future work on ultra-high concentration vertical homo-multijunction solar cells:

- To improve conversion efficiency, research could focus on developing new and improved materials with higher optical and electrical properties.
- Research could focus on optimizing the cell design to reduce resistance losses, improve thermal management, and increase the fill factor.
- An improved concentrator could reduce losses, increase concentration, and reduce the size and cost of the system.
- Research could focus on integrating solar cells with energy storage systems to provide stable, continuous power output and increase the overall efficiency of the system.
- To increase commercial viability and widespread deployment of the system, research could focus on scaling up the production process and reducing the costs of the system.
- The research focus should be on making ultra-high concentration vertical homo-multijunction solar cells a more feasible large-scale energy generation technology by increasing conversion efficiency and reducing costs.

## Figures and Tables

**Table 1:** GaAs VMJ solar cell design and material properties.

Category	Parameter	Value
Thickness	Layer P+ (µm)	0.07
	Layer P (µm)	3–8
	Layer N (µm)	1–6
	Layer N+ (µm)	0.07
Doping	P+ doping (cm^−3^)	5 × 10^19^
	P doping (cm^−3^)	2 × 10^12^–2 × 10^17^
	N doping (cm^−3^)	9 × 10^12^–9 × 10^17^
	N+ doping (cm^−3^)	5 × 10^19^
Connection Region	Layer tunnel N (µm)	0.025
	Layer tunnel_P (µm)	0.025
	N tunnel doping (cm^−3^)	7 × 10^19^
	P tunnel doping (cm^−3^)	7 × 10^19^
	Resistance (Ω · cm^2^)	1.25 × 10^16^
Device Settings	Width (µm)	15.0–20.33
	Sun concentration (sun)	1–10,000
Material Properties	Gap energy (eV)	1.42
	Dielectric Permittivity (ɛ)	13.2
	Affinity χ (eV)	4.07
	Lattice constant (Å)	5.65
	Auger coefficient (cm^6^/s) Doped (n)	1.9 × 10^−31^
	Auger coefficient (cm^6^/s) Doped (p)	12 × 10^−31^

## Data Availability

Data are contained within the article.
